# A decade of glyphosate–surfactant poisoning in India: clinical patterns and predictors of mortality

**DOI:** 10.1186/s12873-026-01696-4

**Published:** 2026-07-22

**Authors:** Sanjan Asanaru Kunju, Ahlam Abdul Rahman, Sachin Nayak Sujir, Vivek Gopinathan, Bhanvi Prakash Ramchandani, Sumedha Sircar

**Affiliations:** 1 Department of Emergency Medicine, Kasturba Medical College Mangalore, Manipal Academy of Higher Education, Manipal, India; 2 Department of Anaesthesiology, Kasturba Medical College Mangalore, Manipal Academy of Higher Education, Manipal, India; 3 Department of Emergency Medicine, Kasturba Medical College, Manipal Academy of Higher Education, Manipal, India; 4 Kasturba Medical College, Manipal Academy of Higher Education, Manipal, India

**Keywords:** Glyphosate, Herbicide poisoning, Mortality predictors, Acute kidney injury, Retrospective study, India

## Abstract

**Background:**

Glyphosate-surfactant herbicide (GlySH) poisoning is increasingly reported in low- and middle-income countries, yet data on clinical predictors of mortality remain limited, particularly from the Indian subcontinent. This study aimed to describe the clinical profile of GlySH poisoning and to identify predictors of in-hospital mortality in a tertiary-care setting in South India.

**Methods:**

We conducted a retrospective observational study of consecutive adult patients with documented oral GlySH ingestion presenting to a tertiary-care emergency department between January 2009 and July 2019. Demographic, clinical, and laboratory variables were extracted from electronic medical records. In-hospital mortality was the primary outcome. Associations between clinical variables and mortality were assessed using Firth’s penalised likelihood logistic regression and Fisher’s exact test, with odds ratios (ORs) and 95% confidence intervals (CIs) reported. For predictors with complete separation, odds ratios were not estimable, and Fisher’s exact p-values are reported. Multivariable analysis was not performed due to the small number of outcome events.

**Results:**

A total of 51 patients were included, of whom four (7.8%) died. Most patients were young adults (45.1% aged 25–44 years), male (76.5%), and had suicidal intent (64.7%). Shock (OR 14.67, 95% CI 1.50–143.74), altered sensorium (OR 14.63, 95% CI 1.34–159.23), elevated serum creatinine > 1.4 mg/dL (*p* = 0.0009), and dialysis requirement (*p* < 0.001) were associated with in-hospital mortality on univariate analysis. Elevated liver transaminases and respiratory distress were not significantly associated with mortality.

**Conclusion:**

In this 10-year cohort, mortality following GlySH poisoning was closely associated with shock, altered sensorium, and early renal dysfunction, reflecting advanced systemic toxicity. These readily identifiable features may assist in risk stratification and triage of high-risk patients in the emergency setting. Given the small number of outcome events, these findings should be interpreted as hypothesis-generating, and larger multicentric prospective studies are required to validate these predictors.

**Supplementary Information:**

The online version contains supplementary material available at https://doi.org/10.1186/s12873-026-01696-4.

## Background

Glyphosate-surfactant herbicides (GlySH) are among the most widely used non-selective systemic herbicides globally, with applications spanning large-scale agriculture, forestry, and domestic weed control. Glyphosate inhibits the shikimate pathway enzyme EPSP synthase, disrupting aromatic amino acid synthesis in plants; in mammals, this pathway is absent, which explains the relatively lower direct toxicity of glyphosate itself. However, commercial formulations contain surfactants — principally polyethoxylated amine, (POEA) which are substantially more toxic than glyphosate alone. POEA disrupts cellular membranes, causes direct mucosal injury, uncouples mitochondrial oxidative phosphorylation, and triggers a systemic inflammatory response, collectively driving haemodynamic collapse, metabolic dysfunction, and multi-organ failure [1–4]. In resource-limited settings, where these herbicides are widely available to the public, this has led to a significant burden of acute poisoning. Mortality in intentional ingestions of concentrated formulations has been reported at up to 30% in some cohorts, with toxicity further determined by the time elapsed before hospital presentation and the surfactant-to-glyphosate ratio in the ingested product [3, 5–7]. Typical clinical manifestations include gastrointestinal corrosive injury, vomiting, diarrhoea, hypotension, pulmonary oedema, acute kidney injury (AKI), arrhythmia, hyperkalaemia, and metabolic acidosis [2, 8]. 

Pesticide-related deaths represent a significant public health burden in resource-limited settings. Although such countries use pesticides less than high-income regions, they account for 99% of fatal pesticide poisoning — a disparity driven by poor regulatory oversight, unsafe storage, widespread household availability, and limited critical-care infrastructure [9, 10]. India presents a particular concern in this regard. Glyphosate-based herbicides are registered under the Insecticides Act 1968 and are commercially available in formulations ranging from 41% soluble liquid (isopropyl amine salt) to 71% soluble granules, sold over the counter in agricultural supply stores with minimal restrictions [11]. Their ready availability, combined with growing herbicide use linked to agricultural mechanisation and declining farm labour, has contributed to a rising incidence of toxic exposures [12]. Local awareness of their toxicity remains insufficient — because glyphosate is branded as a “low-toxicity” product, patients frequently delay seeking medical attention after ingestion.

Most published clinical data originate from East Asia (Taiwan, South Korea, and Japan) where surfactant compositions, formulation strengths, and healthcare systems differ considerably from the Indian context [5, 6, 13]. This heterogeneity raises questions about whether mortality predictors identified internationally — shock, hyperkalaemia, acidosis, and depressed sensorium — are directly applicable to Indian populations, where differences in formulation, prehospital care, and rural presentation delays may produce distinct clinical patterns.

A further challenge is the absence of a validated risk-stratification tool for this poisoning. Unlike paraquat or organophosphate toxicity, for which established prognostic systems exist, clinicians must currently rely on subjective judgement for severity assessment — a gap that is particularly consequential for ED triage and ICU allocation in resource-constrained settings.

This study addresses these gaps by reviewing ten years of herbicide poisoning cases at a South Indian tertiary-care hospital. The aims are to describe the clinical and laboratory profile of patients with documented GlySH ingestion and to identify predictors of in-hospital mortality, with a view to informing emergency triage algorithms and evidence-based risk stratification in similar settings.

## Materials and methods

### Study design and setting

We conducted a retrospective observational study of GlySH poisoning cases at a 1,500-bed tertiary-care teaching hospital in South India, which receives approximately 50,000 visits per year in its Emergency Department (ED). The study included all adult patients documented to have orally ingested GlySH during the period from January 2009 to July 2019. Selection bias was minimised by including all eligible consecutive cases during the study period. Ethical approval was obtained from the Institutional Ethics Committee prior to data abstraction (IEC No. 179/2019; IRB Registration ECR/146/Inst/KA/2013/RR-16; approval date: 7 August 2019). Clinical trial number: not applicable. This study was designed, conducted, and reported in accordance with the Strengthening the Reporting of Observational Studies in Epidemiology (STROBE) guidelines; the completed checklist is provided as Supplementary File 1.

To preserve a consistent pre-COVID-19 cohort and reduce variability introduced by pandemic-related changes to healthcare systems, cases occurring after July 2019 were excluded. As the study is retrospective and utilises anonymised electronic medical records, the requirement for informed patient consent was waived. Data were fully de-identified prior to analysis, and the study complied with institutional and international ethical regulations governing the use of human subjects’ data in research.

### Case identification and selection of participants

Eligible participants were adults aged 18 years and older with documented oral ingestion of GlySH formulations. Potential cases were identified using the International Classification of Diseases, Tenth Revision (ICD-10) discharge code T60 within the hospital electronic medical record (EMR) system. As ICD-10 coding is not specific to GlySH exposures, each identified record was manually reviewed using a structured case identification algorithm. A case was classified as documented GlySH ingestion if: oral ingestion was recorded in the clinical notes; there was no documented co-exposure to other pesticides; and at least one of the following was satisfied — (i) the product label was visually confirmed to contain glyphosate; (ii) a commercial brand known to contain glyphosate was documented; or (iii) the container or brand name was presented and confirmed by a caregiver. Analytical laboratory confirmation of glyphosate was not available, consistent with standard practice in our setting.

We acknowledge that ICD-10 T60 coding compliance was not formally audited during the study period; patients miscoded or assigned alternative diagnostic codes may not have been identified, potentially resulting in under-ascertainment of cases. This is acknowledged as a limitation.

Exclusion criteria were - Uncertain exposure history; co-ingestion of other pesticides; non-oral route of exposure; discharge against medical advice; transfer to another healthcare facility; age under 18 years; and missing essential information. Missing essential information was defined as the absence of survival status, or absence of at least one definitional clinical variable (blood pressure, serum creatinine, or level of consciousness). Paediatric cases were excluded owing to differences in toxicokinetics, weight-based dosing, airway and renal physiology, and management protocols that necessitate separate evaluation.

### Data abstraction and quality assurance

Data abstraction was performed by two emergency medicine residents under faculty supervision using a standardised data collection form. Abstractors received training on study objectives, EMR navigation, standard operating procedures, and a de-identified pilot case prior to commencing data collection. Data were extracted in duplicate to improve reliability, with discrepancies resolved by consensus or adjudication by a senior faculty investigator. As outcome status was available in discharge records, blinding to outcomes was not feasible; to minimise bias, abstractors adhered to pre-defined variable definitions and recorded objective clinical and laboratory values obtained within the first 24 h of ED presentation. For all laboratory parameters, the first available value obtained within 24 h was used for analysis. For vital sign-based definitions such as shock, the sustained threshold criterion was applied as described below. Inter-rater agreement was assessed in a random 10% sample of records, and discrepancies were resolved by consensus with a senior investigator. Regular data audits were conducted to ensure completeness and accuracy.

### Patient management and clinical protocols

All patients were managed according to a standardised institutional protocol aligned with international toxicology and critical-care guidelines, including recommendations from the American Academy of Clinical Toxicology (AACT), the European Association of Poisons Centres and Clinical Toxicologists (EAPCCT), and the EXTRIP workgroup [14–17]. Activated charcoal was administered only within one hour of ingestion in patients with a protected airway; routine gastric lavage was avoided. Where vasopressor support was required, norepinephrine was used as the first-line agent. Haemodialysis was performed exclusively for standard metabolic indications — refractory hyperkalaemia, severe metabolic acidosis, or oliguric AKI — and not for the purpose of toxin removal [17, 18]. 

### Data variables and exposure assessment

Demographic variables collected included age, sex, occupation, marital status, exposure intent (suicidal, accidental, or occupational), estimated ingestion volume, and time from ingestion to ED arrival (categorised as < 6 h, 7–12 h, or > 12 h). Clinical variables included presenting symptoms, vital signs, and laboratory parameters obtained within the first 24 h. Treatment prior to ED arrival (including any interventions administered at referring facilities or primary health centres before presentation to the study hospital, such as gastric lavage, intravenous fluids, or symptomatic care) and in-hospital interventions were recorded, along with need for mechanical ventilation, renal replacement therapy, vasopressor support, ICU admission, length of hospital stay, and survival status.

Ingested volume was recorded using standardised descriptive units adapted from prior literature: spoon (5 mL), mouthful (25 mL), cup (100 mL), and bottle (300 mL) [5]. As volume estimates were self-reported in broad categories and only four deaths occurred, formal dose–response modelling was not feasible. Nausea and vomiting were analysed as a composite variable in comparative analysis.

### Operational definitions of outcomes

In-hospital mortality following GlySH ingestion was the primary outcome. Secondary outcomes were need for mechanical ventilation, need for renal replacement therapy, requirement for vasopressor support, and length of hospital stay. Shock, altered sensorium, and AKI were analysed as predictor variables in the univariate analysis rather than as outcomes.

Shock was defined as systolic blood pressure < 90 mmHg or MAP < 65 mmHg sustained for ≥ 30 min, or requirement for vasopressors after adequate fluid resuscitation, with clinical evidence of hypoperfusion [19]. Altered sensorium was defined as any new impairment in consciousness attributable to poisoning, including Glasgow Coma Scale (GCS) ≤ 13, a ≥ 2-point decline in GCS, AVPU scores of P or U, or documented confusion, agitation, or coma [20, 21]. Patients with post-sedation symptoms within six hours of the last sedative dose were excluded from this definition.

AKI was defined using KDIGO criteria: urine output < 0.5 mL/kg/h for ≥ 6 h, a rise in serum creatinine ≥ 0.3 mg/dL within 48 h, or ≥ 1.5-fold increase from baseline within three months [22]. Early renal dysfunction was defined as the presence of AKI or a serum creatinine > 1.4 mg/dL documented within the first 24 h of ED presentation. The threshold of > 1.4 mg/dL corresponds to the upper limit of the normal adult reference range at our institution’s laboratory and was used as a pragmatic single time-point severity marker, analysed separately from KDIGO-based AKI criteria [23]. When baseline creatinine was unavailable, the lowest in-hospital value was used as reference. Arrhythmia was defined as any rhythm other than normal sinus rhythm documented on ECG. Conservative management referred to supportive care without dialysis or mechanical ventilation.

Acid-base parameters were available only in a subset of patients. pH and bicarbonate measurements were available for approximately half the cohort, and complete acid-base data were available for only two of the four non-survivors. Although these variables were reviewed, the extent of missing data and the very small number of outcome events precluded reliable statistical evaluation. To avoid biased estimates based on incomplete data, metabolic acidosis was not included in the primary analysis. As metabolic acidosis is a recognised prognostic marker in GlySH toxicity, its absence from this analysis is acknowledged as a limitation; future prospective studies should include arterial blood gas analysis as a mandatory data point.

## Sample size and effect size justification

All consecutive eligible cases over the ten-year period were included (*N* = 51; deaths = 4). Sample size was determined by case availability rather than formal power calculation. Multivariable regression analysis was not performed given only four deaths, to prevent model overfitting and unstable estimates. The analysis focused on reporting effect sizes with 95% confidence intervals to describe the strength and precision of associations rather than formal hypothesis testing. Given the sparse outcome events, all statistical associations should be interpreted with caution as exploratory and hypothesis-generating.

### Statistical analysis

Statistical analysis was performed using IBM SPSS Statistics Version 26.0. Continuous variables were tested for normality and summarised as mean ± standard deviation (SD) or median (interquartile range, IQR) as appropriate. Categorical variables were summarised as frequencies and percentages and compared using the chi-square or Fisher’s exact test. Given the small number of outcome events (*n* = 4), Firth’s penalised likelihood logistic regression was applied to determine associations between predictor variables and mortality, providing bias-reduced odds ratio (OR) estimates with 95% confidence intervals (CIs) for all predictors with non-zero cells. For predictors with complete separation (zero cells) — elevated serum creatinine and dialysis requirement — Fisher’s exact test with exact p-values is reported and odds ratios are noted as not estimable. A p-value of < 0.05 was considered statistically significant; however, given the limited number of outcome events, effect sizes and confidence intervals are emphasised over p-values, and causal inferences cannot be drawn.

## Results

### Characteristics of study subjects

**Fig. 1:**
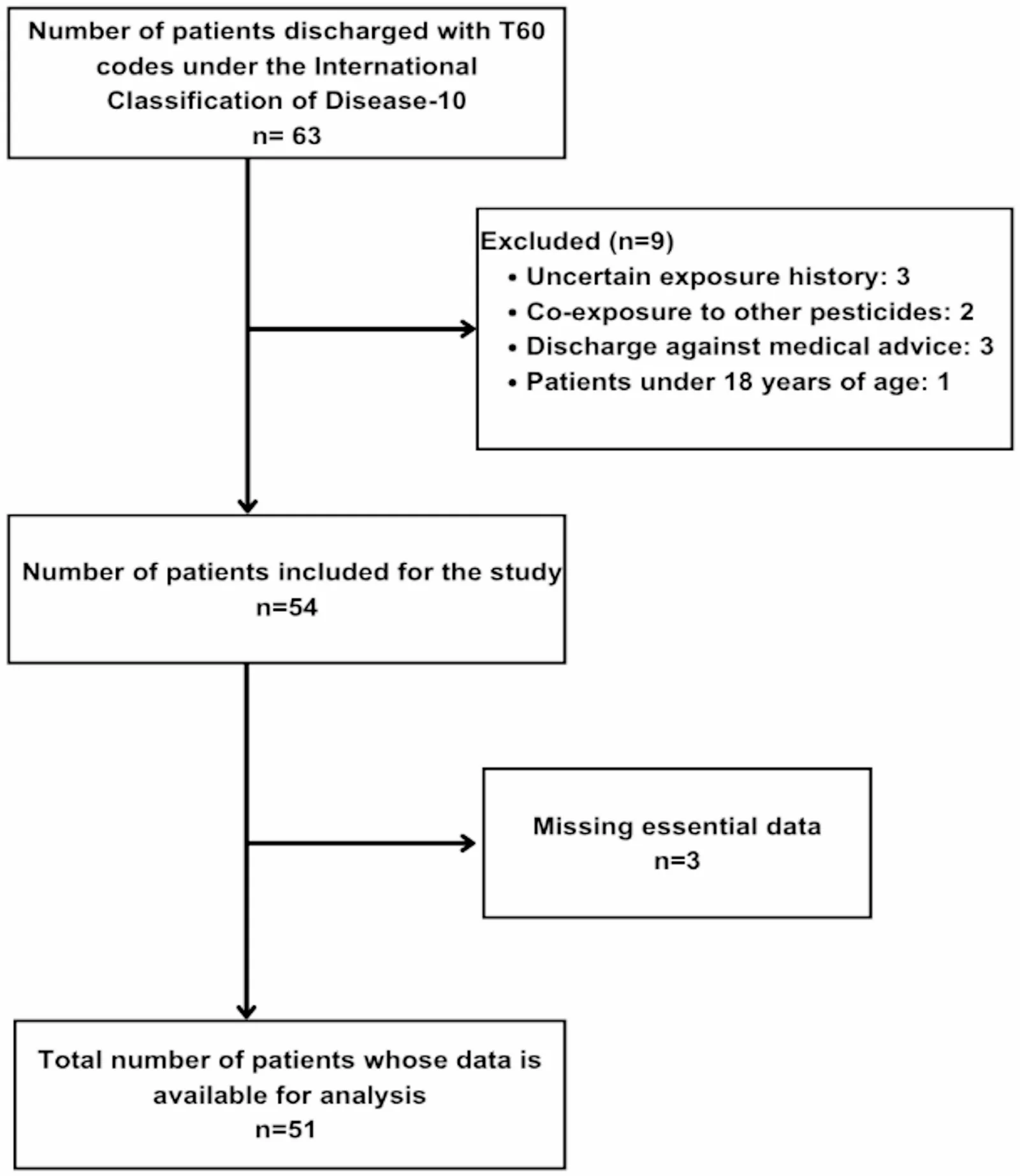
STROBE flow diagram depicting patient identification, exclusion, and final cohort selection

Our initial patient identification yielded 63 patients with ICD-10 T60 discharge codes. Nine patients were excluded: three had uncertain exposure histories, two had co-exposure to other pesticides, three were discharged against medical advice, and one was under 18 years of age. Three patients had essential data missing and were excluded from the final analysis. Following application of exclusion criteria, 51 patients were included in the final analysis. (Fig. 1).

### Baseline demographic and clinical characteristics

The study cohort included 51 patients with documented GlySH ingestion (Table 1). Most patients were aged 25–44 years (45.1%), the majority were male (76.5%), agricultural workers constituted 29.4% of cases, and most patients were married (70.6%). Suicidal intent was reported in 64.7% of exposures.

**Table 1:** Demographic characteristics of GlySH poisoning

Demographic Characteristic	n (%), *n* = 51
Age Range	
18–24 years	20 (39.2%)
25–44 years	23 (45.1%)
45–64 years	6 (11.8%)
65 + years	2 (3.9%)
Age, years -Median (P25–P75)	33 (22–42)
**Gender**	
Male	39 (76.5%)
Female	12 (23.5%)
**Occupation**	
Agriculture	15 (29.4%)
Student	10 (19.6%)
Labourer	8 (15.7%)
Business	4 (7.8%)
Unemployed	14 (27.5%)
**Marital Status**	
Married	36 (70.6%)
Unmarried	15 (29.4%)
**Intent of Exposure**	
Suicidal	33 (64.7%)
Stress-related	9 (17.6%)
Unknown	9 (17.6%)
**Ingested Amount (volumetric units)**	
A spoon (5 mL)	8 (15.7%)
A mouthful (25 mL)	10 (19.6%)
A cup (100 mL)	20 (39.2%)
A bottle (300 mL)	6 (11.8%)
Missing data	7 (13.7%)
**Elapsed Time (hours)**	
< 6	16 (31.4%)
7–12	17 (33.3%)
> 12	10 (19.6%)
Missing data	8 (15.7%)
Elapsed time, hours - Median (P25–P75)	7(5-12)
**Clinical manifestation**	
Nausea	35 (68.6%)
Vomiting	36 (70.6%)
Diarrhoea	5 (9.8%)
Abdominal Pain	11 (21.6%)
Sore throat	7 (13.7%)
Dysphagia	2 (3.9%)
Haematemesis	3 (5.9%)
Oral ulcers	2 (3.9%)
Altered sensorium	11 (21.6%)
Excessive salivation	2 (3.9%)
Chest pain	2 (3.9%)
Fever	2 (3.9%)
Syncope	2 (3.9%)
**Treatment Modality**	
Treatment prior to hospital arrival	38 (74.5%)
Gastric Lavage	41 (80.4%)
Activated Charcoal	4 (7.8%)
Conservative management	45 (88.2%)
Mechanical Ventilation	5 (9.8%)
Dialysis	5 (9.8%)

Abbreviation: GlySH, Glyphosate-surfactant herbicide; mL, Milliliter

Common presenting symptoms included vomiting (70.6%), nausea (68.6%), abdominal pain (21.6%), altered sensorium (21.6%), and fever (3.9%). Fever was defined as a recorded temperature ≥ 38.0 °C within the first 24 h of presentation. Shock was present in 9.8% and respiratory distress in 19.6% of patients. The overall in-hospital mortality rate was 7.8% (4/51). (Table 1)

Treatment prior to ED arrival was documented in 74.5%(*n* = 38) of patients, including gastric lavage at referring facilities in 86.8% and intravenous fluids or symptomatic care in the remainder. In-hospital management was conservative in 88.2% of patients. Mechanical ventilation was required in 9.8% and dialysis in 9.8% of cases. (Table 1)

### Comparison between survivors and non-survivors

Comparisons between survivors (*n* = 47) and non-survivors (*n* = 4) are shown in Table 2. Non-survivors were older than survivors (median 42.5 vs. 32.0 years), though this did not reach statistical significance (*p* = 0.12). There was no significant difference in sex distribution (*p* > 0.99) or treatment prior to ED arrival (*p* = 0.10) between groups.

**Table 2:** Clinical characteristics and outcomes in patients with GlySH toxicity: A comparative analysis of survivors and non-survivors

	Total (*n* = 51)	Survivors (*n* = 47)	Deceased (*n* = 4)	*p-value
Age in years (Median, IQR)	33.0 (21.5–42.0)	32.0 (21.0–41.5)	42.5 (36.8–50.5)	0.12
Gender Distribution (male/female)	39/12	36/11	3/1	> 0.99
Treatment prior to arrival (Yes/No)	38/13	35/12	3/1	0.10
**Symptom**				
Abdominal Pain, n (%)	11 (21.6%)	9 (19.1%)	2 (50.0%)	0.20
Nausea/Vomiting, n (%)	47 (92.2%)	43 (91.5%)	4 (100.0%)	> 0.99
Fever, n (%)	2 (3.9%)	1 (2.1%)	1 (25.0%)	0.152
**Intervention/Complication**				
Mechanical Ventilation, n (%)	5 (9.8%)	4 (8.5%)	1 (25.0%)	0.35
Dialysis, n (%)	5 (9.8%)	1 (2.1%)	4 (100.0%)	< 0.001
Shock, n (%)	5 (9.8%)	3 (6.4%)	2 (50.0%)	0.04
Altered Sensorium, n (%)	11 (21.6%)	8 (17.0%)	3 (75.0%)	0.026
Respiratory Distress, n (%)	10 (19.6%)	8 (17.0%)	2 (50.0%)	0.15
Suicide Attempt	33 (64.7%)	29 (59.6%)	4 (100.0%)	0.28
**Length of hospital stay**,				
Days, Median (IQR)	5.5 (4.0–8.0)	5.0 (4.0–8.0)	13.0 (10.2–18.2)	0.034

Abbreviation: GlySH, Glyphosate-surfactant herbicide; IQR, Interquartile range

*Age and length of hospital stay compared using the Mann–Whitney U test; all other comparisons by Fisher’s exact test

Among presenting symptoms, fever was more frequent among non-survivors than survivors (25% vs. 2.1%, *p* = 0.152) although the difference was not statistically significant. Abdominal pain and nausea/vomiting were more frequent among non-survivors but did not differ significantly between groups. Shock (50.0% vs. 6.4%, *p* = 0.04) and altered sensorium (75.0% vs. 17.0%, *p* = 0.026) were significantly more common among non-survivors. Respiratory distress did not differ significantly between groups. (Table 2)

Ingested volume was self-reported in broad descriptive categories and was missing in 13.7% of cases (*n* = 7). Given the small number of deaths (*n* = 4) and the proportion of missing data, formal comparison of ingested volume between survivors and non-survivors was not performed, as any statistical comparison would be unreliable. The distribution of ingested volume categories for the full cohort is reported in Table 1.

The median length of hospital stay for the entire cohort was 5.5 days (IQR 4.0–8.0), ranging from 1 to 31 days. Survivors had a significantly shorter hospital stay compared with non-survivors (median 5.0 days, IQR 4.0–8.0 vs. median 13.0 days, IQR 10.2–18.2; *p* = 0.034).

### Laboratory Parameters and mortality

Initial laboratory parameters are summarised in Table 3. There were no significant differences in haemoglobin, white blood cell count, or platelet count between survivors and non-survivors. Among biochemical parameters, serum albumin (median 3.9 vs. 4.5 g/dL, *p* = 0.007) and total protein levels (median 6.4 vs. 7.3 g/dL, *p* = 0.024) were significantly lower among non-survivors. Direct bilirubin levels were significantly higher among non-survivors (median 1.1 vs. 0.2 mg/dL, *p* = 0.009), whereas total bilirubin did not differ significantly between groups. There was no statistically significant difference in AST or ALT between survivors and non-survivors. Serum potassium levels did not differ significantly between groups (*p* = 0.06).

**Table 3:** Comparison of initial haematological and biochemical parameters between survivors and deceased patients in GlySH Toxicity

Parameter	Total (*n* = 51), Median (IQR)	Survivors, *n* = 47, Median (IQR)	Deceased, *n* = 4, Median (IQR)	*p*-value
Haematological Parameters				
Haemoglobin (g/dL)	14.2 (13.4–14.9)	14.2 (13.5–14.9)	14.1 (13.5–14.4)	0.89
WBC Count (10^9^/L)	12.1 (11.3–13.5)	12.1 (11.3–13.4)	13.1 (12.7–13.5)	0.74
Platelet Count (10³/mm³)	259 (248–269)	259 (248–269)	259 (256–263)	0.98
**Biochemical Parameters**				
Total Bilirubin (mg/dL)	0.7 (0.4–1.0)	0.6 (0.4–0.9)	1.7 (1.1–4.5)	0.56
Direct Bilirubin (mg/dL)	0.2 (0.2–0.4)	0.2 (0.2–0.3)	1.1 (0.4–2.3)	0.009
Albumin (g/dL)	4.5 (4.1–4.9)	4.5 (4.2–4.9)	3.9 (3.3–4.1)	0.007
Total Protein (g/dL)	7.2 (6.7–7.6)	7.3 (6.8–7.6)	6.4 (5.7–6.7)	0.024
AST (IU/L)	40 (28–54)	39 (28–53)	49 (34–81)	0.73
ALT (IU/L)	28 (18–45)	27 (18–44)	42 (33–61)	0.18
ALP (IU/L)	77 (60–104)	76 (59–103)	80 (62–108)	0.83
Electrolytes				
Sodium (mEq/L)	139 (136–142)	139 (136–142)	137 (135–139)	0.43
Potassium (mEq/L)	4.2 (3.9–4.6)	4.2 (3.9–4.5)	4.9 (4.3–5.5)	0.06
Serum Creatinine (mg/dL)	0.90(0.70–1.30)	0.90(0.70–1.10)	7.80 (5.43–9.93)	0.0014

All comparisons by Mann–Whitney U test

Abbreviations: GlySH, Glyphosate-surfactant herbicide; IQR, Interquartile range; g/dL, grams per deciliter; WBC, white blood cell count; mg/dL, milligrams per deciliter; IU/L, International Units Per Liter

### Univariate predictors of mortality

Firth’s penalised likelihood logistic regression analysis is shown in Table 4. Elevated serum creatinine > 1.4 mg/dL was present in all four non-survivors and was associated with mortality on univariate analysis (Fisher’s exact *p* = 0.0009). An odds ratio was not estimable due to complete separation. Altered sensorium (OR 14.63, 95% CI 1.34–159.23; *p* = 0.026) and shock (OR 14.67, 95% CI 1.50–143.74; *p* = 0.041) were also associated with mortality. Given that only four deaths occurred, confidence intervals are wide, and all associations should be interpreted as exploratory.

**Table 4:** Predictors of mortality and clinical outcomes in GlySH poisoning

Predictors	Survivors, n/N (*N* = 47)	Deceased, n/N (*N* = 4)	Total, n/N, (%) (*N* = 51)	Odds Ratio (95% CI)	*p*-value	Methods
Elevated Creatinine (> 1.4 mg/dL)	4/47	4/4	8 (8/51, 15.7%)	Not estimable	0.0009	Fisher exact
Elevated AST (>40 IU/L)	22/47	3/4	25 (25/51, 49.0%)	3.41 (0.333–35.030)	0.73	Firth
Elevated ALT (>42 IU/L)	7/47	2/4	9/51, (17.6%)	5.71 (0.60–54.69)	0.18	Firth
Dialysis	1/47	4/4	5/51 (9.8%)	Not estimable	<0.001	Fisher exact
Shock	3/47	2/4	5/51 (9.8%)	14.67 (1.50–143.74)	0.041	Firth
Altered Sensorium	8/47	3/4	11/51 (21.6%)	14.63 (1.34–159.23)	0.026	Firth
Respiratory Distress	8/47	2/4	10/51 (19.6%)	4.88 (0.49–48.18)	0.15	Firth

OR estimated using Firth’s penalised likelihood logistic regression except where noted. †OR not estimable due to complete separation (zero cell); Fisher’s exact p-value reported for elevated creatinine and dialysis. All estimates should be interpreted with caution; only four in-hospital deaths occurred, resulting in wide confidence intervals throughout.

Abbreviations: GlySH, Glyphosate-surfactant herbicide; mg/dL, milligrams per deciliter; U/L, Units Per Liter

All four non-survivors required dialysis compared with one survivor (Fisher’s exact *p*<0.001); however, given that dialysis was prescribed exclusively for metabolic indications in the most severely ill patients, this association likely reflects disease severity rather than an independent prognostic effect. Given that only four deaths occurred, this study was powered to detect only very large effect sizes; smaller true differences, if they exist, would not have been detectable. AST, ALT, and respiratory distress were not significantly associated with mortality. (Table 4)

## Discussion

In this 10-year retrospective cohort of patients with documented GlySH ingestion, shock, altered sensorium, and early renal dysfunction were associated with in-hospital mortality on univariate analysis. These findings are consistent with markers of advanced systemic toxicity and should be interpreted as exploratory given the small number of outcome events and the wide confidence intervals that result from sparse data.

The demographic profile was predominantly of young adult males aged 25–44 years engaged in agriculture or unskilled labour, consistent with reports from Korean, Sri Lankan, Taiwanese, and Malaysian cohorts where occupational exposure and easy herbicide accessibility contribute to ingestion risk [24–27]. The occurrence of poisoning across non-agricultural occupational groups indicates that access extends beyond farming communities. In contrast to some international cohorts, age and sex were not significant predictors of mortality, most likely owing to the limited number of deaths, which substantially constrains statistical power to detect demographic associations. The high prevalence of suicidal intent — observed in nearly two-thirds of patients — is consistent with global reports identifying GlySH poisoning as a common means of attempted self-harm, reinforcing the importance of community education, improved mental health care access, and regulatory control of herbicide availability as prevention strategies [9, 10]. 

The clinical spectrum was heterogeneous, encompassing predominantly gastrointestinal manifestations alongside atypical features such as altered sensorium and excessive salivation [28]. Fever was more frequent among non-survivors and may represent an early systemic inflammatory response, aspiration-related infection, or developing sepsis in patients with high-grade toxicity; however, this observation is based on small numbers and requires prospective validation. Hypoalbuminaemia and reduced total protein — markers of systemic inflammation and capillary leak — were significantly lower among non-survivors, while early renal dysfunction reflected the severity of haemodynamic compromise. Liver transaminases did not differ significantly between groups, and hepatic injury was not identified as a mortality predictor in this cohort, consistent with Roberts et al. [28]. Renal dysfunction was the strongest association with mortality, consistent with Korean and Sri Lankan reports, underscoring the prognostic importance of early kidney injury in this population [25, 27]. The three predictors — shock, altered sensorium, and elevated serum creatinine — should be interpreted as markers of advanced systemic toxicity rather than independent causal determinants of death.

The mechanisms underlying these predictors are well characterised in the experimental literature. Shock is primarily driven by POEA-mediated disruption of cellular membrane integrity and mitochondrial uncoupling, resulting in vasoplegia, capillary leak, and distributive circulatory failure — confirmed both in vitro in endothelial cell models and in vivo in swine haemodynamic studies [29–31]. Altered sensorium reflects a combination of cerebral hypoperfusion secondary to haemodynamic instability, direct surfactant neurotoxicity, and metabolic derangements including acidosis and hyperkalaemia; elevated serum S100 protein — a marker of neuronal injury — has been reported in affected patients, suggesting direct central nervous system involvement [32]. AKI is thought to result from renal hypoperfusion in the context of distributive shock compounded by direct tubular toxicity of surfactant components. Importantly, in clinical observational studies it is not possible to disaggregate the individual toxic contributions of glyphosate and its surfactant co-formulants; the findings reported here reflect the composite toxicity of the ingested formulation rather than either component in isolation.

Most patients improved with conservative management, illustrating the value of supportive therapy for mild-to-moderate poisoning. Non-survivors had a significantly prolonged hospital stay compared with survivors (median 13 vs. 5 days, *p* = 0.034), reflecting the resource-intensive clinical course in patients requiring organ support. All four non-survivors required dialysis compared with one survivor (*p* <0.001); however, as dialysis was initiated exclusively for severe metabolic derangements —refractory hyperkalaemia, metabolic acidosis, or oliguric AKI — in the most critically ill patients, this association likely represents a marker of advanced systemic toxicity rather than an independent causal determinant of death. Our findings reinforce the EXTRIP principle that haemodialysis should be prescribed on the basis of standard metabolic indications rather than for the purpose of toxin removal [18, 33]. 

These findings carry particular relevance for South Indian emergency and resource-limited settings, where laboratory turnaround times at primary and secondary care facilities are often prolonged, limiting timely biochemical risk stratification. Clinician awareness of the potential for rapid haemodynamic deterioration following GlySH ingestion remains suboptimal, partly because glyphosate is widely perceived as a low-toxicity herbicide, which may contribute to delayed escalation of care and underutilisation of critical care resources. The absence of a validated local risk-stratification protocol means that triage decisions depend on individual clinical judgement rather than objective criteria, and intensive care capacity at district hospital level is often limited. The three predictors identified — shock, altered sensorium, and elevated serum creatinine — are assessable at the bedside or with basic laboratory infrastructure, and their presence should prompt immediate escalation, close monitoring, and early transfer to a facility with critical care capability where required.

### Limitations

This study has several important limitations inherent to its retrospective single-centre design. Incomplete or inconsistent medical records may have introduced information bias, with potential underreporting or misclassification of key variables including ingested volume, time to presentation, and clinical parameters. As outcome status was available in discharge records, blinding of data abstractors to outcomes was not feasible, which may have introduced ascertainment bias despite the use of pre-defined variable definitions.

Several data completeness gaps further limit the analysis. Ingested volume was self-reported in broad descriptive categories with missing data in 13.7% of cases, precluding reliable comparison between survivors and non-survivors. ICD-10 T60 coding compliance was not formally audited; patients assigned alternative diagnostic codes may not have been captured, potentially resulting in case under-ascertainment. Arterial blood gas data were insufficiently recorded to permit systematic analysis of metabolic acidosis, a recognised prognostic marker in GlySH toxicity that should be routinely collected in future prospective studies.

The dataset spans January 2009 to July 2019 and may not reflect the current spectrum of GlySH formulations available on the Indian market, as surfactant compositions and glyphosate acid equivalent concentrations in commercial products evolve over time. Findings should therefore be interpreted in the context of the formulations prevalent during the study period.

Selection and spectrum bias are likely, given that our cohort was drawn from a tertiary referral centre and may overrepresent more severe presentations. The small number of outcome events (*n* = 4 deaths) substantially limited statistical power, precluded multivariable modelling, and produced wide confidence intervals; all associations should be interpreted as exploratory and hypothesis-generating rather than definitive. Residual confounding from unmeasured variables — including exact ingested dose, formulation composition, baseline comorbidities, and treatment delays — could not be fully addressed. Only short-term in-hospital outcomes were assessed, and long-term renal, hepatic, or neurological sequelae require prospective evaluation. The absence of analytical glyphosate quantification precluded dose–response analyses, and the single-centre design limits generalisability to other regions with differing formulations, healthcare access, and exposure patterns.

## Conclusion

This 10-year retrospective cohort study demonstrates that although most patients with documented GlySH ingestion recover with supportive care, in-hospital mortality is associated with shock, altered sensorium, and early renal dysfunction, reflecting advanced systemic toxicity. These readily identifiable clinical and biochemical features may assist in early risk stratification and triage of high-risk patients in emergency and resource-limited settings.

Given the small number of outcome events and the retrospective design, these findings should be interpreted as hypothesis-generating rather than definitive. Larger multicentric prospective studies with formulation-specific toxicological data are required to validate these predictors and to develop standardised risk-stratification tools for clinical practice.

## Electronic supplementary material

Below is the link to the electronic supplementary material.

Supplementary Material 1

## Data Availability

All data used is available upon request from the corresponding author, other than the supplementary material provided.

## Abbreviations

AKI: Acute kidney injury
ALP: Alkaline phosphatase
ALT: Alanine aminotransferase
AST: Aspartate aminotransferase
AVPU: Alert, voice, pain, unresponsive
ABC: Airway, breathing, circulation
AACT: American academy of clinical toxicology
ABG: Arterial blood gas
CI: Confidence Interval
CIBRC: Central insecticides board and registration committee
COVID-19: Coronavirus disease 2019
DAMA: Discharge against medical advice
ECG: Electrocardiogram
ED: Emergency department
EMR: Electronic medical record
EAPCCT: European association of poisons centres and clinical toxicologists
EPSP: 5-Enolpyruvylshikimate-3-Phosphate
EXTRIP: Extracorporeal treatments in poisoning
GCS: Glasgow coma scale
GlySH: Glyphosate-surfactant herbicide
ICD-10: International classification of diseases, tenth revision
ICU: Intensive care unit
IEC: Institutional ethics committee
IQR: Interquartile range
IRB: Institutional review board
KDIGO: Kidney disease: improving global outcomes
LMICs: Low- and middle-income countries
MAP: Mean arterial pressure
MV: Mechanical ventilation
OR: Odds ratio
POEA: Polyethoxylated amine
RRT: Renal replacement therapy
SBP: Systolic blood pressure
SD: Standard deviation
SPSS: Statistical package for the social sciences
STROBE: Strengthening the reporting of observational studies in epidemiology

## References

[1] Seok SJ, Park JS, Hong JR, Gil HW, Yang JO, Lee EY, et al. Surfactant volume is an essential element in human toxicity in acute glyphosate herbicide intoxication. Clin Toxicol. 2011;49(10):892–9. 10.3109/15563650.2011.626422 22077202

[2] Williams GM, Kroes R, Munro IC. Safety evaluation and risk assessment of the herbicide Roundup and its active ingredient, glyphosate, for humans. Regul Toxicol Pharmacol. 2000;31(2):117–65. 10.1006/rtph.1999.1371 10854122

[3] Amrhein N, Deus B, Gehrke P, Steinrücken HC. The site of the inhibition of the shikimate pathway by glyphosate: II. Interference of glyphosate with chorismate formation in vivo and in vitro. Plant Physiol. 1980;66(5):830–4. 10.1104/pp.66.5.830 PMC440735 16661535

[4] Bradberry SM, Proudfoot AT, Vale JA. Glyphosate poisoning. Toxicol Rev. 2004;23:159–67. 10.2165/00139709-200423030-00003 15862083

[5] Goldstein DA, Acquavella JF, Mannion RM, Farmer DR. An analysis of glyphosate data from the California Environmental Protection Agency pesticide illness surveillance program. J Toxicol Clin Toxicol. 2002;40(7):885–92. 10.1081/clt-120016960 12507058

[6] Székács A, Darvas B. Forty years with glyphosate. Herbic-Prop Synth Control Weeds. 2012;14:247–84.

[7] Beswick E, Millo J. Fatal poisoning with glyphosate-surfactant herbicide. J Intensive Care Soc. 2011;12(1):37–9.

[8] Jeyaratnam J, Chia K. Occupational health in national development. World Scientific; 1994.

[9] Gunnell D, Eddleston M. Suicide by intentional ingestion of pesticides: a continuing tragedy in developing countries. Int J Epidemiol. 2003;32(6):902–9. 10.1093/ije/dyg307 PMC2001280 14681240

[10] World Health Organization. International code of conduct on pesticide management. Guidance on use of pesticide regulation to prevent suicide. World Health Organization; 2023.

[11] Central Insecticides Board and Registration Committee (CIBRC), Directorate of Plant Protection, Quarantine & Storage, Ministry of Agriculture and Farmers Welfare, Government of India. Insecticides/Pesticides registered under Sect. 9(3) of the Insecticides Act 1968. New Delhi: Government of India. 2023. Available from: https://ppqs.gov.in/sites/default/files/9._molecules_registered_under_section-93_as_on_01.06.2023.pdf

[12] Banerjee I, Tripathi S, Roy AS, Sengupta P. Pesticide use pattern among farmers in a rural district of West Bengal, India. J Nat Sci biology Med. 2014;5(2):313. 10.4103/0976-9668.136173 PMC4121905 25097405

[13] Tominack RL, Tominack R. Herbicide formulations. J Toxicol Clin Toxicol. 2000;38(2):129–35. 10.1081/clt-100100927 10778909

[14] Benson B, Hoppu K, Troutman W, Bedry R, Erdman A, Höjer J, et al. Position paper update: gastric lavage for gastrointestinal decontamination. Clin Toxicol. 2013;51(3):140–6. 10.3109/15563650.2013.770154 23418938

[15] Zellner T, Prasa D, Färber E, Hoffmann-Walbeck P, Genser D, Eyer F. The use of activated charcoal to treat intoxications. Dtsch Aerzteblatt Int. 2019;116(18):311. 10.3238/arztebl.2019.0311 PMC6620762 31219028

[16] Bouchard J, Roberts DM, Roy L, Ouellet G, Decker BS, Mueller BA. et al. Principles and operational parameters to optimize poison removal with extracorporeal treatments. Wiley Online Libr; 2014. pp. 371–80. 10.1111/sdi.12247 24824059

[17] Ghannoum M, Roberts DM, Hoffman RS, Ouellet G, Roy L, Decker BS et al. A stepwise approach for the management of poisoning with extracorporeal treatments. Wiley Online Libr; 2014. pp. 362–70. 10.1111/sdi.12228 24697864

[18] Garlich FM, Goldman M, Pepe J, Nelson L, Allan M, Goldstein D, et al. Hemodialysis clearance of glyphosate following a life-threatening ingestion of glyphosate-surfactant herbicide. Clin Toxicol. 2014;52(1):66–71. 10.3109/15563650.2013.870344 24400933

[19] Evans L, Rhodes A, Alhazzani W, Antonelli M, Coopersmith CM, French C, et al. Surviving sepsis campaign: international guidelines for management of sepsis and septic shock 2021. Crit Care Med. 2021;49(11):e1063–143. 10.1097/CCM.0000000000005337 34605781

[20] American College of Emergency Physicians. Clinical policy for the initial approach to patients presenting with altered mental status. Ann Emerg Med. 1999;33(2):251–81. 10.1016/s0196-0644(99)70406-3 14765552

[21] Zuercher M, Ummenhofer W, Baltussen A, Walder B. The use of Glasgow Coma Scale in injury assessment: a critical review. Brain Inj. 2009;23(5):371–84. 10.1080/02699050902926267 19408162

[22] Kidney Disease. Improving Global Outcomes (KDIGO) AKI Work Group. KDIGO clinical practice guideline for acute kidney injury. Kidney Int Suppl. 2012;2(1):1–138.

[23] Levey AS, Coresh J, Balk E, et al. National Kidney Foundation practice guidelines for chronic kidney disease: evaluation, classification, and stratification. Ann Intern Med. 2003;139(2):137–47. 10.7326/0003-4819-139-2-200307150-00013 12859163

[24] Chen YJ, Wu ML, Deng JF, Yang CC. The epidemiology of glyphosate-surfactant herbicide poisoning in Taiwan, 1986–2007: a poison center study. Clin Toxicol. 2009;47(7):670–7. 10.1080/15563650903140399 19640238

[25] Cha ES, Khang YH, Lee WJ. Mortality from and incidence of pesticide poisoning in South Korea: findings from National Death and Health Utilization Data between 2006 and 2010. PLoS ONE. 2014;9(4):e95299. 10.1371/journal.pone.0095299 PMC3990630 24743877

[26] Kamaruzaman NA, Leong YH, Jaafar MH, Khan HRM, Rani NAA, Razali MF, et al. Epidemiology and risk factors of pesticide poisoning in Malaysia: a retrospective analysis by the National Poison Centre (NPC) from 2006 to 2015. BMJ Open. 2020;10(6):e036048. 10.1136/bmjopen-2019-036048 PMC7265005 32487578

[27] Van Der Hoek W, Konradsen F. Risk factors for acute pesticide poisoning in Sri Lanka. Trop Med Int Health. 2005;10(6):589–96. 10.1111/j.1365-3156.2005.01416.x 15941423

[28] Roberts DM, Buckley NA, Mohamed F, Eddleston M, Goldstein DA, Mehrsheikh A, et al. A prospective observational study of the clinical toxicology of glyphosate-containing herbicides in adults with acute self-poisoning. Clin Toxicol. 2010;48(2):129. 10.3109/15563650903476491 PMC2875113 20136481

[29] Song HY, Kim YH, Seok SJ, Gil HW, Yang JO, Lee EY, et al. Cellular toxicity of surfactants used as herbicide additives. J Korean Med Sci. 2012;27(1):3–9. 10.3346/jkms.2012.27.1.3 PMC3247771 22219606

[30] Lee HL, Kan CD, Tsai CL, Liou MJ, Guo HR. Comparative effects of the formulation of glyphosate-surfactant herbicides on hemodynamics in swine. Clin Toxicol. 2009;47(7):651–8. 10.1080/15563650903158862 19663613

[31] Moon JM, Chun BJ, Cho YS, Lee SD, Hong YJ, Shin MH, et al. Cardiovascular effects and fatality may differ according to the formulation of glyphosate salt herbicide. Cardiovasc Toxicol. 2018;18:99–107. 10.1007/s12012-017-9418-y 28612304

[32] Lee Jwon, Choi Y, jin, Park S, Gil HW, Song H, yeon, Hong SY. Serum S100 protein could predict altered consciousness in glyphosate or glufosinate poisoning patients. Clin Toxicol. 2017;55(5):357–9. 10.1080/15563650.2017.1286013 28301275

[33] Mi Moon J, Il Min Y, Jo Chun B. Can early hemodialysis affect the outcome of the ingestion of glyphosate herbicide? Clin Toxicol. 2006;44(3):329–32. 10.1080/15563650600584550 16749554

