# Supplementary material for: A decade of glyphosate–surfactant poisoning in India: clinical patterns and predictors of mortality
Source: BMC Emerg Med. 2026 Jul 22;26:276. doi: 10.1186/s12873-026-01696-4 (PMC13685134; doi:10.1186/s12873-026-01696-4)
Supplement: Supplementary file 1 [file 12873_2026_1696_MOESM1_ESM.docx]

**STROBE Checklist — Cohort Study**

**Manuscript: A Decade of Glyphosate–Surfactant Poisoning in India: Clinical Patterns and Predictors of Mortality**

| **Item** | **Section & Topic** | **Done?** | **Recommendation (Cohort Study)** | **Location in Manuscript** |
| --- | --- | --- | --- | --- |
| **TITLE AND ABSTRACT** | | | | |
| **1** | **Title & Abstract** | **Yes** | (a) Indicate the study design with a commonly used term in the title or abstract.  (b) Provide an informative and balanced summary of what was done and what was found. | (a) Title: 'A Decade of Glyphosate–Surfactant Poisoning in India: Clinical Patterns and Predictors of Mortality'. Abstract Methods: 'We conducted a retrospective observational study...'  (b) Abstract provides structured summary: Background, Methods, Results (n=51, 4 deaths, ORs with 95% CIs reported), Conclusion. Page 1. |
| **INTRODUCTION** | | | | |
| **2** | **Background / Rationale** | **Yes** | Explain the scientific background and rationale for the investigation being reported. | Introduction, Paragraphs 1–5: global burden of GlySH poisoning, POEA mechanism, Indian regulatory context (Insecticides Act 1968, 41–71% w/v), gap in regional mortality data, absence of validated risk-stratification tool. Pages 2–3. |
| **3** | **Objectives** | **Yes** | State specific objectives, including any prespecified hypotheses. | Introduction, Final paragraph: 'The aims are to describe the clinical and laboratory profile of patients with documented GlySH ingestion and to identify predictors of in-hospital mortality, with a view to informing emergency triage algorithms and evidence-based risk stratification in similar settings.' Page 3. |
| **METHODS** | | | | |
| **4** | **Study Design** | **Yes** | Present key elements of study design early in the paper. | Methods, Study Design and Setting, first sentence: 'We conducted a retrospective observational study of GlySH poisoning cases at a 1,500-bed tertiary-care teaching hospital in South India...' Page 3. |
| **5** | **Setting** | **Yes** | Describe the setting, locations, and relevant dates, including periods of recruitment, exposure, follow-up, and data collection. | Methods, Study Design and Setting: 1,500-bed tertiary-care teaching hospital, South India; approximately 50,000 ED visits/year; study period January 2009 to July 2019; IEC approval 7 August 2019. Page 3. |
| **6** | **Participants** | **Yes** | (a) Give the eligibility criteria, sources and methods of selection of participants, and methods of follow-up.  (b) For matched studies, give matching criteria and number of exposed and unexposed. (Not applicable here.) | (a) Methods, Case Identification: Inclusion — adults ≥18 years, oral GlySH ingestion, January 2009–July 2019. Exclusion — uncertain history, co-ingestion, non-oral route, DAMA, transfer, age <18, missing essential data. Documented using structured case identification algorithm applied to ICD-10 T60 records. Pages 3–4.  (b) N/A — no matched design used. |
| **7** | **Variables** | **Yes** | Clearly define all outcomes, exposures, predictors, potential confounders, and effect modifiers. Give diagnostic criteria if applicable. | Methods, Operational Definitions of Outcomes: Primary outcome (in-hospital mortality), secondary outcomes (MV, RRT, vasopressor support, LOS), predictor variables (shock, altered sensorium, AKI) all formally defined with criteria and citations. Shock: SBP <90 or MAP <65 for ≥30 min or vasopressor requirement (Ref 25). Altered sensorium: GCS ≤13 or AVPU P/U (Refs 26–27). AKI: KDIGO criteria (Ref 28). Creatinine >1.4 mg/dL: upper limit of normal reference range (Ref 29). Pages 5–6. |
| **8** | **Data Sources / Measurement** | **Yes** | For each variable of interest, give sources of data and details of methods of assessment. Describe comparability of assessment methods if there is more than one group. | Methods, Data Abstraction and Quality Assurance: EMR-based extraction by two trained residents using standardised data collection form; duplicate extraction; discrepancies by consensus with senior faculty; first available lab value within 24 hours used; inter-rater agreement assessed on 10% random sample. Page 4. |
| **9** | **Bias** | **Yes** | Describe any efforts to address potential sources of bias. | Methods: consecutive case inclusion to minimise selection bias; pre-defined variable definitions to reduce ascertainment bias; blinding not feasible — acknowledged in Data Abstraction paragraph and Limitations. ICD-10 coding compliance gap acknowledged as limitation. Page 4 and Limitations section, Page 11. |
| **10** | **Study Size** | **Yes** | Explain how the study size was arrived at. | Methods, Sample Size and Effect Size Justification: 'All consecutive eligible cases over the ten-year period were included (N=51; deaths=4). Sample size was determined by case availability rather than formal power calculation. Multivariable regression analysis was not performed given only four deaths.' Page 6. |
| **11** | **Quantitative Variables** | **Yes** | Explain how quantitative variables were handled in the analyses. If applicable, describe which groupings were chosen and why. | Methods, Statistical Analysis: continuous variables tested for normality, summarised as mean ± SD or median (IQR) as appropriate. Serum creatinine dichotomised at >1.4 mg/dL — rationale and citation provided (Ref 29). Age reported in predefined 10-year categories in Table 1; as continuous median (IQR) in Table 2. Page 6. |
| **12** | **Statistical Methods** | **Yes** | (a) Describe all statistical methods, including those used to control for confounding.  (b) Describe any methods used to examine subgroups and interactions.  (c) Explain how missing data were addressed.  (d) If applicable, explain how loss to follow-up was addressed.  (e) Describe any sensitivity analyses. | (a) Methods, Statistical Analysis: Fisher's exact test, chi-square test, Mann–Whitney U test, Firth's penalised likelihood logistic regression for predictors with non-zero cells; Fisher's exact test for predictors with complete separation (zero cells — elevated creatinine and dialysis). IBM SPSS v26.0. Page 6.  (b) Survivors vs non-survivors subgroup comparison throughout Tables 2, 3, 4. No interaction terms given n=4 deaths.  (c) Ingested volume missing in 13.7% (n=7) — reported in Results and Limitations; group comparison not performed. Elapsed time missing in 15.7% (n=8) — noted in Table 1.  (d) N/A — retrospective in-hospital study; no post-discharge follow-up.  (e) N/A — sensitivity analyses not performed given n=4 outcome events; acknowledged as limitation. |
| **RESULTS** | | | | |
| **13** | **Participants** | **Yes** | (a) Report numbers at each stage of study.  (b) Give reasons for non-participation at each stage.  (c) Consider use of a flow diagram. | (a) Results, Characteristics of Study Subjects: 63 identified by ICD-10 T60; 9 excluded (3 uncertain history, 2 co-ingestion, 3 DAMA, 1 age <18); 3 missing essential data; 51 in final analysis. Page 7.  (b) Reasons for each exclusion category stated in Results paragraph. Page 7.  (c) Figure 1: STROBE flow diagram depicting patient identification, exclusion, and final cohort selection. Page 7. |
| **14** | **Descriptive Data** | **Yes** | (a) Give characteristics of study participants and information on exposures and potential confounders.  (b) Indicate number of participants with missing data for each variable of interest.  (c) Summarise follow-up time. | (a) Table 1: full demographic, clinical, and treatment characteristics of all 51 patients. Table 2: comparison by survivor/non-survivor status. Pages 13–15.  (b) Ingested volume missing n=7 (13.7%) stated in Results; elapsed time missing n=8 (15.7%) in Table 1. Elapsed time median reported as 8 hours (P25–P75: 4–12 hours), estimated from grouped categorical data.  (c) N/A — in-hospital retrospective study; no post-discharge follow-up period. |
| **15** | **Outcome Data** | **Yes** | Report numbers of outcome events or summary measures over time. | Results, Baseline Characteristics: 'The overall in-hospital mortality rate was 7.8% (4/51).' Secondary outcomes: mechanical ventilation 9.8% (5/51), dialysis 9.8% (5/51), conservative management 88.2% (45/51). LOS: median 5.5 days (IQR 4.0–8.0). Page 7. |
| **16** | **Main Results** | **Yes** | (a) Give unadjusted estimates and their precision (95% CI). Make clear which confounders were adjusted for.  (b) Report category boundaries when continuous variables were categorised.  (c) If relevant, translate estimates of relative risk into absolute risk. | (a) Table 4: Firth's penalised likelihood logistic regression ORs with 95% CIs and p-values for all predictors with non-zero cells. For predictors with complete separation (creatinine, dialysis): OR not estimable, Fisher's exact p reported. Multivariable analysis not performed — rationale stated. Creatinine: p=0.0009. Shock: OR 14.67 (1.50–143.74), p=0.041. Altered sensorium: OR 14.63 (1.34–159.23), p=0.026. Page 16.  (b) Serum creatinine dichotomised at >1.4 mg/dL — rationale and citation provided in Methods (Ref 29).  (c) N/A — not applicable to this exploratory retrospective study with sparse outcome data. |
| **17** | **Other Analyses** | **Yes** | Report other analyses done — e.g. analyses of subgroups and interactions, and sensitivity analyses. | Results: full survivor vs non-survivor comparison for all demographic, clinical, and laboratory variables in Tables 2 and 3. LOS comparison (Mann–Whitney U, p=0.034) in Results and Table 2.  Sensitivity analyses not performed; acknowledged as limitation in Limitations section, Page 11. |
| **DISCUSSION** | | | | |
| **18** | **Key Results** | **Yes** | Summarise key results with reference to study objectives. | Discussion, opening paragraph: 'In this 10-year retrospective cohort of patients with documented GlySH ingestion, shock, altered sensorium, and early renal dysfunction were associated with in-hospital mortality on univariate analysis.' Directly references stated objectives. Page 9. |
| **19** | **Limitations** | **Yes** | Discuss limitations of the study, taking into account sources of potential bias or imprecision. Discuss both direction and magnitude of any potential bias. | Limitations section (4 paragraphs, Pages 11–12): retrospective design, information bias, ascertainment bias, ICD-10 coding compliance, missing ingested volume data (13.7%), ABG data gap, dataset age (2009–2019), selection/spectrum bias (tertiary referral), small sample (n=4 deaths), wide CIs, no multivariable modelling, residual confounding, no long-term follow-up, no analytical toxicological confirmation, single-centre generalisability. |
| **20** | **Interpretation** | **Yes** | Give a cautious overall interpretation of results considering objectives, limitations, multiplicity of analyses, results from similar studies, and other relevant evidence. | Discussion: associations described as 'exploratory' and 'hypothesis-generating' throughout. Comparisons drawn with Korean, Sri Lankan, Taiwanese, and Malaysian cohorts (Refs 13, 30–32). Mechanistic interpretation of findings provided. South India contextual paragraph added (Pages 10–11).  Conclusion: 'findings should be interpreted as hypothesis-generating rather than definitive.' Page 12. |
| **21** | **Generalisability** | **Yes** | Discuss the generalisability (external validity) of the study results. | Limitations, final paragraph: 'the single-centre design limits generalisability to other regions with differing formulations, healthcare access, and exposure patterns.' Page 12.  Discussion, South India paragraph: contextualised applicability to resource-limited Indian settings specifically discussed. Pages 10–11. |
| **OTHER INFORMATION** | | | | |
| **22** | **Funding** | **Yes** | Give the source of funding and the role of the funders for the present study and, if applicable, for the original study on which the present article is based. | Declarations, Source(s) of Support and Funding: 'The authors report that no external funding or support was received for this article.' Page 12. |
